# Supplementary material for: Mechanisms governing the pioneering and redistribution capabilities of the non-classical pioneer PU.1
Source: Nat Commun. 2020 Jan 21;11:402. doi: 10.1038/s41467-019-13960-2 (PMC6972792; doi:10.1038/s41467-019-13960-2)
Supplement: Supplementary file 7 — Source data [file 41467_2019_13960_MOESM7_ESM.zip › Source_Data/Figure6/Figure6B_MotifScanOutput/ETS1_PU.1/homerResults/motif16.similar.html]

motif16

## Information for motif16

T
C
G
A
A
C
G
T
A
C
T
G
C
G
T
A
T
A
C
G
A
C
G
T
G
T
A
C
C
G
T
A
A
C
G
T
A
G
T
C
  
Reverse Opposite:  

T
C
A
G
T
G
C
A
A
C
G
T
A
C
T
G
C
G
T
A
A
T
G
C
A
C
G
T
G
T
A
C
C
G
T
A
A
G
C
T
  

|  |  |
| --- | --- |
| p-value: | 1e-143 |
| log p-value: | -3.304e+02 |
| Information Content per bp: | 1.792 |
| Number of Target Sequences with motif | 1313.0 |
| Percentage of Target Sequences with motif | 8.29% |
| Number of Background Sequences with motif | 920.0 |
| Percentage of Background Sequences with motif | 2.87% |
| Average Position of motif in Targets | 101.4 +/- 52.8bp |
| Average Position of motif in Background | 103.8 +/- 60.0bp |
| Strand Bias (log2 ratio + to - strand density) | 0.0 |
| Multiplicity (# of sites on avg that occur together) | 1.05 |
| Motif File: | file (matrix) reverse opposite |

### Similar de novo motifs found

|  |  |  |  |  |  |  |  |
| --- | --- | --- | --- | --- | --- | --- | --- |
| Rank | Match Score | Redundant Motif | P-value | log P-value | % of Targets | % of Background | Motif file |
| 1 | 0.963 | A C G T A C T G G T C A A T C G A C G T G T A C C T G A A G C T | 1e-141 | -326.026608 | 10.05% | 4.00% | motif file (matrix) |
| 2 | 0.966 | A G T C C T A G T C G A C A G T A C T G G C T A T A C G C G A T G T A C G C T A A C G T | 1e-140 | -322.417601 | 8.51% | 3.05% | motif file (matrix) |
| 3 | 0.975 | G A C T A C T G G T C A A T C G A C G T T G A C C T G A A C G T G A T C | 1e-138 | -319.131603 | 9.36% | 3.60% | motif file (matrix) |
| 4 | 0.913 | A C G T A C T G G T C A A C T G A C G T A G T C C G T A | 1e-137 | -317.091009 | 11.09% | 4.75% | motif file (matrix) |
| 5 | 0.954 | C T G A C A T G C A T G T C G A C G A T A C T G C G T A T A C G A C G T A T G C C G T A A C G T | 1e-135 | -311.061491 | 7.76% | 2.67% | motif file (matrix) |
| 6 | 0.940 | A C T G C T A G T G A C A C G T A C T G C G T A A C T G A C G T A G T C C G T A A G T C G A T C C A G T | 1e-121 | -280.665033 | 5.70% | 1.65% | motif file (matrix) |
